# Federalism, an alternative to overcome the inequalities of sustainable development in the natural regions and macro regions of Peru

**DOI:** 10.3389/fsoc.2023.1219310

**Published:** 2023-07-26

**Authors:** Mario Aurelio Coyla Zela, José Luis Morales Rocha, Teófilo Lauracio Ticona, Jarol Teófilo Ramos Rojas, Gregorio Arroyo Japura, Nakaday Irazema Vargas Torres

**Affiliations:** ^1^School of Public Management and Social Development, National University of Moquegua, Moquegua, Peru; ^2^School of Accounting, Jose Carlos Mariátegui University, Moquegua, Peru

**Keywords:** sustainable development, natural regions, macro regions, GDP per capita, HDI, marginal population, corruption index

## Abstract

Political doctrines determine the formation of territorial units or geopolitical models more adequate to generate public value in subnational organizations, with positive results in the HDI, productivity indicators and national competitiveness oriented to improve the quality of public service for citizens who participate in democratic electoral processes with identity and that promote a decentralized State that does not generate development obstacles as an effect of geographical limits by departments and regional governments with inequalities in natural resources and comparative advantages, but that projects integration, better economic performance, sustainability, and sustainability for regional and national development and as an alternative to achieve political stability in Peru. The aim of this article is to explore whether the contribution of natural regions and macro-regions to sustainable development is unequal. Information from official web pages. The disparity index, analysis of variance (ANOVA) and Tukey's analysis were used. The GDP per capita of the coastal departments is 64% higher than that of the Andes and 136% higher than those of the Amazon; the HDI of the coast exceeds those regions by 31 and 19%; 44% of the urban population residing in marginal neighborhoods are on the coast, 67% in the Andes and 69% in the Amazon. The percentage of corruption is highest on the coast, followed by the Amazon. At the regional macro level, the primacy in GDP per capita and the HDI moves to the south, although the superiority of the corruption index persists in the center, followed by the south; both significantly higher than the east and north. This fact would reveal the possibility of a regional macro development without Lima and through axes of various development nodes, feasible in the era of globalization.

## 1. Introduction

Ansolabehere and Puy (2022), proposes a model of endogenous constitutional design in a scenario where culturally and economically distinct regions establish a national union and negotiate on the form of government. It posits two scenarios: the type of legislative decision rule (consensus or majority rule) and the power of the central government over member regions (centralized or decentralized federalism). His results show that more centralized regimes emerge when regions are culturally similar e.g., the same language or religion and when the economic benefits derived from the union are high. These two aspects of democratic governance of federal societies have clear implications for overall economic growth and the durability of the polity. He concludes that the choice of legislative decision rule and the degree of centralization are integrally linked.

In Gohwong (2021), the findings showed that the governments of Thailand and Argentina used a set of policy instruments and blockchain as a financial innovation to promote their balances and survival of their political systems. In addition, both countries used active and passive measures to maintain the stability of their political systems. They used all the aforementioned policy instruments and innovative CBDCs to change the configuration of the public sector to maintain the equilibrium of their political systems in accordance with Easton (1965) political system model. Public policy demonstrates the course of government action or inaction in the provision of public services to correct market and government failures.

In Burret et al. (2022), they point out that fiscal federalism can be both a resource and a threat to economic performance, even more so when measured by a single variable such as fiscal decentralization. In their methodological contribution, they emphasize that not only fiscal federalism should be considered, but also the study of decentralization, the degree of fiscal competition, jurisdictional fragmentation or the design of its allocation or control.

The territory refers to the spatial structure, while territoriality refers to the process of transformation of nature in tune with the evolution of the human habitat (Herrera Montero, 2020). In the Andean culture, the territory is the source of life, it is the mythical Pachamama, which must be cared for and improved. This vision is coherent with the social construction of territoriality, an approach that demands participatory governance of the agents that inhabit a territory, even those that are in transit or are linked by productive, commercial, financial or other activities. All of them contribute visions, knowledge and ways of appropriating the space (Cohen et al., 2022).

The human habitat compatible with the environment translates into the quality of public spaces, the networks of basic services, the location of the houses, among others; guaranteeing respect for the human right to access, transit and remain in a place, in a safe, comfortable and autonomous manner. The quality of life of citizens is conditioned by the quality of the public space where they usually carry out their activities, where opportunities for meeting, social relations, and contact with nature are created (García, 2019). Apparently, the country is far from guaranteeing the exercise of this right, 21.8% of the Peruvian population that lives in rural areas, lacks full access to basic services such as drinking water, drainage, electricity, telephony, internet, educational centers, health centers, recreational spaces, etc.; to which must be added the population of the marginal neighborhoods, between 25 to 75% of the urban population, the majority distributed in the departments of the Andes and the Amazon. This marginality is usually attributed to internal migrations, which shaped the current urban situation of Lima and the main cities, several of them capitals of the Sanchez (2017) and Seminario et al. (2019); but keep in mind that these migrations are the ones that boosted labor supply and demand, goods and services, public and private finances, idiosyncrasy, culture, political relevance, among others, of the town of your new residence, weakening those of its origin.

Social geography makes it possible to significantly explain regional development in Peru (Vilca et al., 2021). Along the same lines (Roncal and Liza, 2015) state that natural advantages are associated with geography, whether due to proximity to raw materials or population concentration as a source of market and labor. However, the unsustainable use of natural resources, instead of triggering regional or local growth, causes an adverse effect, as occurs with irresponsible mining. In recent decades, Latin American countries, including Peru, have experienced significant economic growth, but social and territorial inequalities persist, it could be said that they are even more perceptive; For example, there are more social conflicts of environmental origin, the disparity of sustainable development between the departments of the country is maintained or tends to increase (Fernández-Labbé, 2022; INEI, 2023c).

Apparently, the social construction of the territories of the regions is uneven, mainly as a result of the location of the headquarters of public institutions, commercial and financial activities, educational and health services, the size of the market whose reference is the population density, among others. In this understanding, metropolitan Lima with a third of the population, the three cities with one million inhabitants and four of the nine cities with half a million inhabitants, tip the balance to the best social construction of the territory of the natural region to which they belong, in this case the coast. This appreciation could be extended to the corresponding macro region, although metropolitan Lima does not usually radiate its star to its surroundings.

In a long-standing investigation (Seminario et al., 2019) they found that the natural regions of the country: the coast, the Andes or the Amazon affect the GDP of the departments located in them; They also showed that the disparity is structural, since the probability of a poor department in 1795 continuing to be so in 2017 is 94%, and that a rich region in that distant era continues to be so today is 95%. Also, that the incidence of the geographical factor is not the same throughout the space and horizon of analysis.

There is a need to evaluate the most appropriate geopolitical model to generate greater public value of subnational organizations, higher levels of competitiveness and productivity aimed at improving the quality of public service to citizens based on a democracy with a democratic electoral system, a decentralized State that does not generate obstacles to development promoted by geographical boundaries by departments and regional governments with inequality in natural resources, but rather projects integration for regional and national development (Aroney, 2008).

The objective of this article is to explore whether the social construction of natural regions: coast, the Andes and the Amazon; and in the macro regions: north, center, south and east show their unequal contribution to sustainable development, expressed in the indicators that were assumed to be more sensitive such as GDP per capita, HDI, percentage of the urban population that resides in marginal neighborhoods and the corruption of the departments of Peru.

## 2. Method

**Figure 1:**
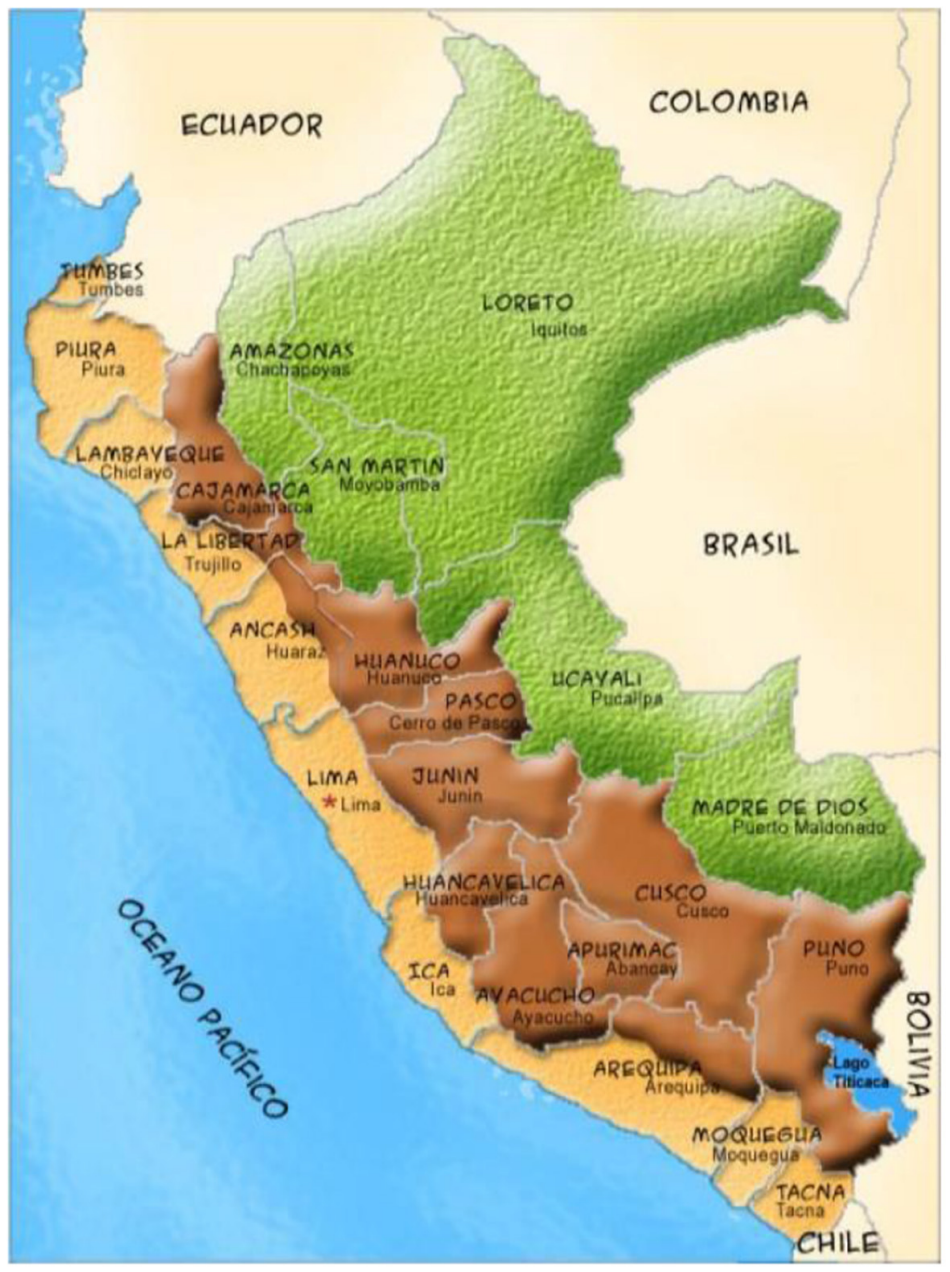
Geographical and political division of Peru.

The scope of study is made up of the 25 departments into which the country is politically divided, including the Constitutional Province of Callao, which has that category (see Figure 1). Eleven departments have their head on the coast of the Pacific Ocean, nine are Andean and five Amazonian. They are disparate in terms of geographic extension, population, urbanism, productive vocation, productivity, market size, industrialization, globalization, competitiveness, educational offer, among others; although it is likely to find similarities between the departments located in those geographical regions (Seminario et al., 2019), as well as between those that make up the macro regions, in addition to the geographical context; with the exception of the center due to the presence of the metropolitan Lima macrocephalic. Five departments are from the north, nine from the center, seven from the south and four from the east. Table 1 presents the relationship of departments by geographic regions and macro regions.

**Table 1:** Sustainable development indicators of the natural regions and macro regions of Perú.

**Natural region/ macro region**	**Departments**	**GDP 2021 base price 2007**		**Population 2021**		**GDP per capita 2021**	**HDI 2019**	**% Population slums 2017**	**% Corruption 2020**
		**Thousands of suns**	**%**	**Amount**	**%**				
Coast	La Libertad, Lambayeque, Piura, Tumbes, Ancash, Callao, Ica, Lima, Arequipa, Moquegua, Tacna	390,726,272	78.2	21,959,316	66.5	18.9	0.591	44.0	3.6
The Andes	Cajamarca, Ayacucho, Huancavelica, Huánuco, Junín, Pasco, Apurímac, Cusco, Puno	84,584,925	16.9	7,915,659	24.0	11.5	0.453	65.7	2.3
Amazon	Madre de Dios. Amazonas, Loreto, San Martín, Ucayali	24,634,439	4.9	3,160,329	9.6	8.0	0.496	71.8	2.8
Total country		499,945,636	100.0	33,035,304	100.0				
North	La Libertad, Lambayeque, Piura, Tumbes, Cajamarca	71,559,031	14.3	7,162,400	21.7	10.0	0.515	59.3	2.2
Center	Ancash, Callao, Ica, Lima, Ayacucho, Huancavelica, Huánuco, Junín, Pasco	319,205,643	63.8	17,579,061	53.2	14.9	0.525	54.6	3.6
South	Arequipa, Moquegua, Tacna, Apurímac, Cusco, Puno, Madre de Dios	86,284,694	17.3	5,313,202	16.1	19.6	0.556	51.1	3.2
East	Amazonas, Loreto, San Martín, Ucayali	22,896,268	4.6	2,980,641	9.0	7.6	0.467	72.2	2.1
Total country		499,945,636	100.0	33,035,304	100.0				

Source: Own elaboration with data from INEI (2020, 2023a,b,c) and Instituto Peruano de Economía (2023).

The information comes mainly from data published on the portals and official web pages of public and private institutions: these data correspond to the years 2017 to 2021. To evaluate the inequality between the departments of a region, the disparity index (ID = standard deviation/mean). Despite the fact that the design is non-experimental, the analysis of variances (ANOVA) has been used to establish the inequality between regions and the Tukey analysis was used to determine if some regions are superior to others.

## 3. Result

The departments of the coast generated 78.2% of the GDP of 2021, whose population is 66.5% of the country; On the other hand, those from the Andes contribute 16.9% but their population is 24% and those from the Amazon 4.9% and their population is 9.6%. The imbalance of production and population in favor of the departments headquartered in the Pacific Ocean is notorious. The average per capita GDP of the coastal departments is 64% higher than that of the Andes and 136% higher than those of the Amazon; As for the HDI, the coast exceeds 31 and 19% respectively; The opposite occurs in the urban population residing in marginal neighborhoods, these are 44% on the coast, 67% in the Andes and 69% in the Amazon. The percentage of corruption is highest on the coast, followed by the Amazon.

It seems that the geographical factor inevitably influences the disparity of regional and local development, as shown by Seminario et al. (2019). The classic regions of the Coast, Sierra and Montaña, into which the European conquerors divided the territory of Tahuantinsuyo, similar to the Iberian Peninsula, do not correspond to our reality due to the different geographical, biological and cultural variability of the Peruvian geography, preventing a social construction of the more homogeneous territory, as recently proposed by Pulgar Vidal (Vejarano and Morales, 2016). Probably what prevailed in the Spanish to relocate the administrative center of their government to the coast, in particular to Lima, was its proximity to Callao, the main port, as well as Arica and others, through which they shipped the enormous natural resources they extracted from the interior of the country, was consolidated during the Republic. Agriculture and livestock were practiced in the nine municipalities of the viceroyalty, mining was also practiced in Huancavelica and Tarma, and forestry was practiced in Maynas. Manufacturing was a subsidiary activity of Huamanga and other localities in the obraje modality, trade was incipient and the relevant transport was maritime that was carried out through the ports (Seminario et al., 2019).

However, at the beginning of the republican era, Lima and the other cities of the Coast did not have any advantage over the other departments; only 100 years later its economic growth began to slow down, but mainly due to migration (Seminario et al., 2019). This historical evidence recognizes migrants as driving agents of economic disparity. These have reconfigured the current Peruvian society says (Sanchez, 2017), strengthened the town of their new residence, but depopulated the one of their origin. In the two centuries of republican history, relevant economic events occurred that (Seminario et al., 2019) link to the growth or decrease of the gross domestic product, such as the construction of the central railways to transport minerals less valuable than silver. and from the south to transport textile raw materials; the extraction of guano from the islands of the Pacific coast, the extraction of rubber in the Amazon, export agriculture on the north coast and center of the country; At the end of the 19th century, the incipient industrialization of some coastal cities began, gaining importance in the mid-20th century: the GDP of the industry went from 20.2% in 1929 to 27.3% in 1970. During these years, the policy of replacing import, industrial poles and the creation of public companies for this purpose, which were dismantled in the last decade of the 20th century; also the agrarian reform through the expropriation of the latifundia, with whose lands the associative companies were organized, which soon gave way to the smallholding and the significant fall of export agriculture, but rescued the peasant from treatment close to medieval serfdom.

From the perspective of the macro-regions, the north, with a population of just over a fifth of the population, contributes a sixth of the GDP; productivity in terms of GDP is slightly higher in the center and in the south; but it is half in the Amazon. The HDI is higher in the south, followed by the center and the north, while the East is relatively far away. Urban marginality is highest in the Amazon, followed by the north and center, and lowest in the south. Corruption is lower in the departments of Amazonia and the north, but it is the same in the departments of the center and the south (see Table 1). The performance in terms of GDP and HDI of the south is striking, equal to or better than the center; despite the fact that it is home to metropolitan Lima, whose market can be associated with the third of the population that resides in that conglomerate; however, this great city does not radiate its growth and improvement within the department to which it belongs and less to its neighbors; apparently, articulating the axes of development around Lima would not contribute to regional development; but rather promote a decentralized system of these independent axes of the capital of the Republic (Espinoza et al., 2022), the possibility is revealed by the performance of the south.

The inequality between the departments of the same region, that is, the endogenous disparity, with respect to GDP per capita, is greater on the coast, followed by the Andes. The same indicator in the macro-regions is higher in the south, followed by the center; but it is irrelevant in the north and in the Amazon (see Table 2). Indeed, Castillo (2019) found that between 2007 and 2012 all the geographical regions of Peru experienced a decrease in endogenous inequality, but this began to revert from this last year, perhaps because neither the momentum nor the duration were sufficient to counteract the endemic culture of discrimination and inequality. Similarly Seminario and Palomino (2021) they found that the concentration of economic activity until 1936 followed the same trend; Lima became relevant and marked the route from that year. However, from 1980, the economic growth of the regions, with or without Lima, began to run on its own.

**Table 2:** Disparity indices of sustainable development indicators of the natural regions and macro regions of Peru.

**Indicators**	**Natural regions**			**Macro regions**			
	**Coast**	**The Andes**	**Amazon**	**North**	**Center**	**South**	**East**
GDP per capita 2021	0.5	0.3	0.1	0.1	0.3	0.6	0.1
HDI 2019	0.1	0.1	0.1	0.1	0.2	0.2	0.1
% of population in slums 2017	0.3	0.1	0.1	0.1	0.2	0.4	0.1
% corruption 2020	0.6	0.6	0.6	0.4	0.6	0.5	0.3

Source: Own elaboration based on data from INEI (2020, 2023a,b,c) and Instituto Peruano de Economía (2023).

No relevant endogenous disparity is observed in the HDI in any of the natural regions as well as in the macro-regions, with a slight relevance in the center and in the south; On the other hand, there is a disparity in the percentage of the urban population in the marginal neighborhoods in the coastal departments and in the southern macro region. The phenomenon of corruption is heterogeneous and considerable in the departments of all the natural regions and in the macro-regions. Although it is not imperative, economic development is part of human development, it also precedes demographic concentration (Aldeanueva Fernández and Cervantes Rosas, 2019; Seminario and Palomino, 2021); although it is not possible to affirm the same regarding corruption.

The disparity of economic development within the departments of the coast, also those of the southern macro region are relevant: ID 0.5 and 0.6 respectively (Table 2), precisely those that obtained the highest productivity in terms of GDP per capita, 18.9 and 19.6 thousand soles, respectively (Table 1); On the other hand, the inequalities between the departments of the regions with the lowest performance are more homogeneous. This phenomenon also extends to the percentage of the marginal urban population. This leads one to wonder if greater development is accompanied by greater heterogeneity; or if, the presence of focal points does not radiate that development to the regions, even localities, neighboring.

The analysis of the variance of the means of the sustainable development indicators between the natural regions, also between the macro regions, reveals that these are different (see Tables 3, 4). The 2019 GDP per capita of the coast reveals its superiority to that of the Andes and the Amazon, the same occurs with the HDI and with the corruption index; both historically explainable by political, administrative, cultural centralism, migrations of the last century, among others. These results show the relative superiority of economic and human development in the coastal departments; but not so to the practice of morality and ethics, unavoidable conditions for the sustainability of development (UNDOC, 2023).

**Table 3:** Analysis of the variance of the sustainable development indicators in natural regions.

**Indicators**	**ANOVA**		**Means**		
			**Natural regions**		
	***F***	***p*** **-value**	**Costa**	**The Andes**	**Amazon**
GDP per capita 2019	26.06	0.0001	18.9^*^	11.5	8.0
HDI 2019	8.51	0.0024	0.591^*^	0.453	0.496
Population in slums	9.85	0.0008	44.0	65.7	71.7^*^
Corruption (%)	101.9	0.0001	3.6^*^	2.3	2.8

^*^Significant superiority with regard to the rest of the means, accourding to Tukey test.

**Table 4:** Analysis of the variance of the sustainable development indicators in macro regions.

**Indicators**	**ANOVA**		**Macro regions**			
	***F***	***p*** **-value**	**North**	**Center**	**South**	**East**
GDP per capita 2019	19.6	0.0001	9.6	14.9	19.6^*^	7.6
HDI 2019	59.7	0.0001	0.556	0.507	0.556^*^	0.467
Population in slums (%)	33.6	0.0001	60.2.	54.6	51.1	72.2^*^
Corruption (%)	55.1	0.0001	2.00	3.6^*^	3.2^*^	2.08

^*^Significant superiority with regard to the rest of the means, according to Tukey test.

Source: Own elaboration based on data from INEI (2023a,b) and Instituto Peruano de Economía (2023).

The percentage of the urban population residing in marginal neighborhoods is significantly higher in the Amazon, possibly due to recent massive migration to urban centers, far exceeding the capacity and practicality of all urban planning that is often not promoted by local governments in emerging municipalities, due to the scarce source of their own financing.

At the regional macro level, the primacy in GDP per capita and the HDI moves to the south, although the superiority of the corruption index persists in the center, followed by the south; both significantly higher than the east and north. This fact would reveal the possibility of macro-regional development without Lima and through axes of various development nodes, feasible in the era of globalization.

## 4. Discussion

Peru is currently in a situation of political crisis because it does not achieve consensus in the implementation of public policies or these do not have the impacts or sustainability over time, which makes it necessary to establish new strategies for equitable redistribution of economic growth with social impacts for which it is necessary to establish that promote a decentralized state that does not generate obstacles to development as a result of the inadequate distribution of the public budget by geographical boundaries or municipalities, departments and regional governments with inequalities in natural resources and comparative advantages, and whose study and implementation generates integration and presents alternatives to achieve political stability in Peru. In this sense, Ansolabehere and Puy (2022) proposes a model of endogenous constitutional design in a scenario where culturally and economically distinct regions establish a national union and negotiate on the form of government. He posits two scenarios: the type of legislative decision rule and the power of the central government over the member regions. These two aspects of democratic governance of federal societies have clear implications for overall economic growth and the durability of the system of government.

Always as an alternative to overcome the inequalities of sustainable development in the natural and macro regions of Peru, it becomes necessary to apply innovation in political transactions by national agreement with ethical level and equitable detachment in the distribution of wealth for national development with positive social impacts, if trying to maintain the survival of the political system as Gohwong (2021) posits, in which the governments of Thailand and Argentina used a set of policy tools and blockchain as financial innovation to promote their balances and survival of their political systems. In addition, both countries used active and passive measures to maintain the stability of their political systems and focused on implementing public policies to demonstrate the government's course of action or inaction in providing public services to correct market and government failures.

Federalism, due to its economic, social, environmental and institutional autonomy, would make it possible to promote the sustainable development of macro-regions for a transversal geographic integration due to the availability of natural resources, biodiversity, interculturality, inclusion, etc., the federalism model would be applied in a sustainable and sustainable manner, with prospects for an increase in regional and national economic performance. And as Burret et al. (2022) suggests, it is possible to complement fiscal federalism, fiscal decentralization, the degree of fiscal competition, jurisdictional fragmentation or the design of its granting or control.

In the 60s of the last century, that decentralist desire of the inhabitants of the interior of the country was resumed in the face of the oppressive centralism of Lima. The last important attempt began in 2001, the eighth in the republican era, characterized by a scheme of transfer of powers and functions to sub-national governments: municipalities and departments or regions (Congress of the republic, 2002; Herz, 2019). After 60 years, in fact and in the imagination of politicians, strategists, particularly of the population, metropolitan Lima stands as an overwhelming focal point of growth and development on the coast and in the interior of the country. An alternative, undoubtedly in the long term, could be the development of radial axes, far from polarity. An example of this possibility is the performance of the southern macro region. In this there is no single development pole, but rather interconnected axes, whose visible nodes are several cities: Arequipa, with close to a million inhabitants; Cusco, Juliaca, even Puno and Tacna, with a population close to half a million inhabitants. These axes are interconnected with each other, with the productive, commercial and other endogenous systems, but also with the national and international markets, near and far. For example, Puno interconnects its economic, social, political agents, etc., from the different localities of the department; as well as with Arequipa. Cusco, Lima, Tacna, Moquegua, Madre de Dios, etc., on the internal front; with Bolivia, Chile, Brazil, Argentina, etc., on the external front; Like Cusco, it attracts international tourism. Something similar happens with the other departments.

Some activists (Oxfam en Perú, 2022) tend to establish a fifth macro region with metropolitan Lima, due to its macrocephalic status in the national economy, also in politics, in culture, among others; indeed, its dynamics and potentialities are very different from the others, but it coexists with the others and feeds on them. Apparently, the challenge is not to replicate what happens with that department, historically exclusive, not only to the other regions, even with its own provinces; but rather to build axes of sustainable development based on the dynamics and axes of macro-regional development. A first step could be the social reconstruction of the country on the basis of the ecological regions of Pulgar Vidal, leaving aside the division of the country into Coast, Sierra and Selva, imposed in another era.

The dilemma of rebuilding the country through macro-regions or ecological regions or others confronts the high doses of political autonomy of the authorities and citizens of each of the departments, which are also often called regions. Faced with this aspiration, very difficult if not impossible to overcome, Arana Velarde (2016) proposes groups of cities formed gravitationally, in the north Trujillo can lead, in the south Arequipa, in the center Huancayo and in the east Iquitos, compatible with the four macro regions; around which networks of cities and systems of production, marketing, finance, etc. would be articulated, leaving regional and local governments and their territories unscathed. Apparently this approach is another replica of the construction of an economic hub similar to metropolitan Lima, with the difference of its location in the traditional historical macro-regions; In addition, from the perspective of regional macro public governance, it would not achieve a rational, efficient and effective decentralization, oriented toward autonomy framed in endogenous values and resources.

The preceding discussion leads us to rethink an unpleasant idea in the country: federalism. This current dates from the 19th century, concluding with the disruption of the very short-lived Peru-Bolivian Confederation: 1836–1839, made up of three states: North-Peru, South-Peru and Bolivia. Each state had to elect its president and govern itself according to its laws, subject to a central regime. It was dissolved with the defeat of General Santa Cruz, its creator and protector, in the battle of Ingavi (Pighi, 2021). Other attempts were: the federalist uprising of Loreto in 1898: it was reported by the lawyer Torres Lara, reflecting on nature, culture and the Amazonian inhabitants, urging an efficient articulation of the internal border territory (Alcedo, 2022); and, the federalist decentralist movement of southern Peru-Puno, 1915–1920, promoted by characters with a desire for local power.

One hundred years later, the interest in federalism as an alternative to good government seems to flourish in the face of the current inefficiency of the unitary State of Peru, bearing in mind the paternalistic vision of Lima with other regions, justifies that the Homeland can move toward a Federal Republic. so that each region can manage its resources (Fuentes, 2023).

## 5. Conclusions

The average per capita GDP of the coastal departments is 64% higher than that of the Andes and 136% higher than those of the Amazon; As for the HDI, the coast exceeds 31 and 19% respectively; 44% of the urban population residing in marginal neighborhoods are on the coast, 67% in the Andes and 69% in the Amazon. The percentage of corruption is highest on the coast, followed by the Amazon.

In the macro-regions, the north, with a population of just over a fifth of the population, contributes a sixth of the GDP, it is slightly higher in the center and in the south; but it is half in the Amazon. The HDI is higher in the south, followed by the center and the north. Urban marginality is highest in the Amazon, followed by the north and center, and lowest in the south. Corruption is lower in the departments of the Amazon and the north, but it is the same in the departments of the center and the south.

The endogenous disparity, with respect to GDP per capita, is greater on the coast, followed by the Andes. The same indicator in the macro-regions is higher in the south, followed by the center; but it is irrelevant in the north and in the Amazon. No relevant endogenous disparity is observed in the HDI in any of the natural regions as well as in the macro-regions, with a slight relevance in the center and in the south; On the other hand, there is a disparity in the percentage of the urban population in the marginal neighborhoods in the coastal departments and in the southern macro region. The phenomenon of corruption is heterogeneous and considerable in the departments of all the natural regions and in the macro-regions.

At the regional macro level, the primacy in GDP per capita and the HDI moves to the south, although the superiority of the corruption index persists in the center, followed by the south; both significantly higher than the east and north. This fact would reveal the possibility of a regional macro development without Lima and through axes of various development nodes, feasible in the era of globalization.

The interest in federalism as an alternative to good government seems to be greening before the current inefficiency of the unitary State of Peru, bearing in mind the paternalistic vision of Lima with other regions, justifies that the Homeland can advance toward a Federal Republic, so that each region can manage your resources.

## Data availability statement

The raw data supporting the conclusions of this article will be made available by the authors, without undue reservation.

## Author contributions

All authors listed have made a substantial, direct, and intellectual contribution to the work and approved it for publication.
